# Bread and Health

**Published:** 1898-02

**Authors:** 


### Bread and Health.

There is no one thing so important in the family as bread. Bread and butter form a staple food in every household, yet the aim of the miller is to make a flour that shall be perfectly white, no matter how much of the good part of the wheat is eliminated in the process. Comparatively few of the housekeepers of the land know how much has been sacrificed of the most nutritious portions of the wheat kernel to prepare for their consumption a white flour. It would be as sensible to soak a piece of porterhouse steak in hot water to get rid of the color and make it white, as to take the mineral salts and phosphates out of the wheat kernel for the sake of making a white flour. The sooner parents find this out, and the more they use of whole wheat flour, the better for the growing children, who need all the nourishment they can get from their food to knit their young bones together. The whole wheat flour is a trifle more expensive at the beginning than the white flour, but the amount of nourishment secured for every dollar expended is largely in excess of that to be had in white flour.—*Exchange.*

THE late Dr. Frank H. Hamilton is responsible for these pithy points:

"1. The best thing for the inside of a man is the outside of a horse. 2. Blessed is he who invented sleep; but thrice blessed the man who will invent a cure for thinking. 3. Light gives a bronzed or tan color to the skin; but where it uproots the lily it plants the rose. 4. The lives of most men are in their own hands, and, as a rule, the just verdict after death would be—'felo de se.' 5. Health must be earned—it can seldom be bought. 6. A change of air is less valuable than a change of scene. The air is changed every time the wind is changed. 7. Mould and decaying vegetables in a cellar weave shrouds for the upper chambers. 8. Dirt, debauchery, disease and death are succesive links in the same chain. 9. Calisthenics may be very genteel, and romping very ungenteel, but one is the shadow, the other the substance, of healthful exercise. 10. Girls need health as much —nay, more, than boys. They can obtain it as boys do, by running, tumbling—by all sorts of innocent vagrancy. At least once a day girls should have their halters taken off, their bars should be let down, and they should be turned loose like young colts.—*Pacific Drug Review.*

